# The Diagnostic Performance of DCE-MRI in Evaluating the Pathological Response to Neoadjuvant Chemotherapy in Breast Cancer: A Meta-Analysis

**DOI:** 10.3389/fonc.2020.00093

**Published:** 2020-02-12

**Authors:** Qingqing Cheng, Jiaxi Huang, Jianye Liang, Mengjie Ma, Kunlin Ye, Changzheng Shi, Liangping Luo

**Affiliations:** ^1^Medical Imaging Center, The First Affiliated Hospital of Jinan University, Guangzhou, China; ^2^Engineering Research Center of Medical Imaging Artificial Intelligence for Precision Diagnosis and Treatment, Guangzhou, China

**Keywords:** dynamic contrast-enhanced magnetic resonance imaging, breast cancer, neoadjuvant chemotherapy, pathological response, meta-analysis

## Abstract

**Background:** Neoadjuvant chemotherapy (NAC) is commonly utilized in preoperative treatment for local breast cancer, and it gives high clinical response rates and can result in pathologic complete response (pCR) in 6–25% of patients. In recent years, dynamic contrast-enhanced magnetic resonance imaging (DCE-MRI) has been increasingly used to assess the pathological response of breast cancer to NAC. In present analysis, we assess the diagnostic performance of DCE-MRI in evaluating the pathological response of breast cancer to NAC.

**Materials and Methods:** A systematic search in PubMed, the Cochrane Library, and Web of Science for original studies was performed. The Quality Assessment of Diagnostic Accuracy Studies-2 tool was used to assess the methodological quality of the included studies. Patient, study, and imaging characteristics were extracted, and sufficient data to reconstruct 2 × 2 tables were obtained. Data pooling, heterogeneity testing, forest plot construction, meta-regression analysis and sensitivity analysis were performed using Stata version 12.0 (StataCorp LP, College Station, TX).

**Results:** Eighteen studies (969 patients with breast cancer) were included in the present meta-analysis. The pooled sensitivity and specificity of DCE-MRI were 0.80 (95% confidence interval [CI]: 0.70, 0.88) and 0.84 (95% [CI]: 0.79, 0.88), respectively. Meta-regression analysis found no significant factors affecting heterogeneity. Sensitivity analysis showed that studies that set pathological complete response (pCR) (*n* = 14) as a responder showed a tendency for higher sensitivity compared with those that set pCR and near pCR together (*n* = 5) as a responder (0.83 vs. 0.72), and studies (*n* = 14) that used DCE-MRI to early predict the pathological response of breast cancer had a higher sensitivity (0.83 vs. 0.71) and equivalent specificity (0.80 vs. 0.86) compared to studies (*n* = 5) that assessed the response after NAC completion.

**Conclusion:** Our results indicated that DCE-MRI could be considered an important auxiliary method for evaluating the pathological response of breast cancer to NAC and used as an effective method for dynamically monitoring the efficacy during NAC. DCE-MRI also performed well in predicting the pCR of breast cancer to NAC. However, due to the heterogeneity of the included studies, caution should be exercised in applying our results.

## Introduction

With the primary clinical goals of downstaging the disease, improving operability, and allowing breast-conserving surgery, neoadjuvant chemotherapy (NAC) is commonly utilized in preoperative treatment for local breast cancer (1–3). NAC gives high clinical response rates (~80%) and can result in pathologic complete response (pCR) in 6–25% of breast cancer patients (4, 5). A previous meta-analysis (6) of 11,955 samples published in the Lancet in 2015 showed that patients who attain pCR after NAC have improved event-free survival and total survival, i.e., pCR was significantly associated with long-term survival benefits. However, approximately 20% of patients may not benefit clinically or pathologically from NAC. Accurate and timely assessment of the pathological response of NAC can provide guidance for the selection of treatment options for patients. Pathological examination, as the gold standard for tumor response evaluation, has high diagnostic accuracy but must be carried out after surgery, so it is easy to miss the best time to adjust the programme. Therefore, it is highly necessary to find a way to dynamically evaluate the tumor response to NAC *in vivo* without invasion so that the treatment can be adjusted at an earlier time for both responders and non-responders.

Various imaging methods have been utilized to assess the pathological response in patients with breast cancer to NAC, including ultrasonography, mammography, breast magnetic resonance imaging (MRI), and positron emission tomography/computed tomography (PET/CT). Although many studies (7–11) have attempted to determine the optimal diagnostic modes for evaluating the efficacy of NAC, no consensus has been reached. In the last 10 years, dynamic contrast-enhanced magnetic resonance imaging (DCE-MRI) has been increasingly used to assess and early predict the pathological response of breast cancer to NAC (12–16). These studies differ in the assessment parameters of DCE-MRI, the evaluation methods, the magnetic field strengths, the number of patients, the definition of responders, and so an. The findings also vary from study to study. Many studies (12–14, 16, 17) have shown a good diagnostic performance of DCE-MRI in assessing the pathological response of breast cancer to NAC, but the optimal parameters are different in different studies. Many other studies (11, 18, 19) have shown a medium diagnostic accuracy of DCE-MRI. The NCCN guidelines for breast cancer 2019 suggest that MRI examination can help assess the extent of the tumor before and after NAC, the remission status of the treatment, and the feasibility of breast-conserving surgery. However, MRI findings alone cannot determine the surgical approach and further biopsy is required.

In the current meta-analysis, we attempted to evaluate the diagnostic performance of DCE-MRI in assessing the pathological response of breast cancer to NAC and address the heterogeneity among the included studies. Due to the lack of standardization in clinically relevant covariates, a sensitivity analysis can investigate how the time of examination, the definition of pathological response, the magnetic field strength, the study design and the evaluation index variations affect diagnostic performance.

## Materials and Methods

### Literature Search

We formulated the study question for this meta-analysis following patient populations, interventions, comparators, outcomes, and study design (PICOS) criteria (20). The study question was developed as follows: what is the diagnostic performance of DCE-MRI in evaluating the pathological response to NAC in breast cancer, with histopathological results used as the reference standard?

A systematic search in PubMed, the Cochrane library, Web of Science, and Embase for studies assessing the diagnostic performance of DCE-MRI in evaluating the pathological response of breast cancer to NAC was performed. We used a combination of the synonyms for breast cancer, MRI and NAC as a search string. The references of the identified articles were checked to identify additional related studies. The search was performed in February 2019, without a start date limit and was restricted to studies published in English.

### Study Selection

The studies included in our review were required to meet the following PICOS criteria: (1) patients who were diagnosed with breast cancer and had received neoadjuvant chemotherapy; (2) patients who had undergone DCE-MRI to assess the response to NAC before mammectomy or breast-conserving surgery; (3) histopathology used as the reference standard; (4) sufficient data to construct 2 × 2 tables were provided; (5) study was an original article and published in English.

Two reviewers performed the literature search and study selection independently. A third reviewer was invited to discuss and reach a consensus when disagreement arose.

### Data Extraction

We extracted the following data from each included study: (1) patient characteristics: patient number, the definition of pathological responder, number/percentage of responders, patient age; (2) study characteristics: authors, publication year, study design (consecutive or non-consecutive, prospective or retrospective,), whether DCE-MRI was used to early predict the pathological response; (3) imaging characteristics: magnet field strength, response assessment parameters or criteria.

### Data Quality Assessment

We assessed the methodological quality of the included studies by using the Quality Assessment of Diagnostic Accuracy Studies-2 tool (21). The risk of bias and applicability was scored for the following four domains: patient selection, index tests, reference standard, flow and timing. Quality assessment was performed using Review Manager 5.3 software.

### Data Analysis

The primary purpose of the present analysis was to explore the diagnostic accuracy of DCE-MRI to assess the pathological response to NAC in breast cancer. The secondary purpose of the study was to evaluate the heterogeneity among the included studies and explore the potential reasons for the heterogeneity.

We extracted two-by-two (2 × 2) contingency tables containing the true and false positive and negative from all included studies to calculate the sensitivity, specificity, area under the curve (AUC), diagnostic odds ratios (DOR), positive likelihood ratio (PLR), negative likelihood ratio (NLR), and the confidence intervals (CIs). If different imaging parameters of DCE-MRI were studied, we chose the most accurate one to calculate the pooled sensitivity and specificity. If the patients were divided into different groups according to different tumor types or different research models, we studied them as a whole.

The coupled forest plots for the sensitivity and specificity and the summary receiver operating characteristic curve (SROC) were drew. The area under the curve (AUC) was used to summarize the diagnostic accuracy. An AUC close to 1 shows a perfect test, and an AUC close to 0.5 shows a poor test. The heterogeneity of the included studies was explored using Cochran's Q test and the inconsistency index (*I*^2^ value), with *P* < 0.05 or *I*^2^ > 50% indicating the presence of substantial heterogeneity. The threshold effect is an important cause of heterogeneity. To test the presence of the threshold effect, the Spearman correlation coefficients were used. There was no threshold effect with a *P* > 0.05.

Meta-regression analyses of several clinically relevant covariates were performed to study the causes of heterogeneity: the time of examination (whether DCI-MRI was used to early predict the response), the definition of pathological responder (pCR or pCR and near pCR together), magnetic field strength (3.0 or 1.5 T), study design (prospective or retrospective), and evaluation index (different assessment parameters or criteria of DCE-MRI). In addition, sensitivity analyses for the relevant covariates described above were performed to explore the influence of those covariates on pooled sensitivity and specificity estimates.

Deek's test for determining the publication bias was conducted. The statistical analyses were performed using Stata version 12.0 (StataCorp LP, College Station, TX).

## Results

### Literature Search

**Figure 1:**
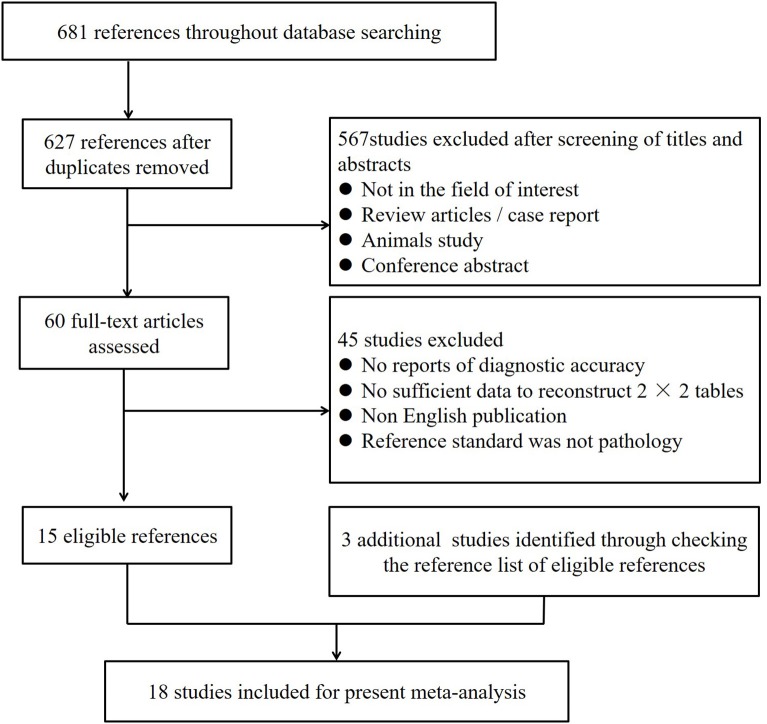
Flow diagram of study selection for meta-analysis.

A total of 681 studies were obtained through electronic searches in PubMed, Cochrane Library, Web of Science, and Embase. Fifty-four articles were removed due to duplication. After a screening of the titles and abstracts, 567 studies were excluded. Full-texts of the remaining 60 studies were reviewed in detail, and an additional 45 studies were excluded for the following reasons: (1) no diagnostic accuracy was reported; (2) no sufficient data were given to reconstruct 2 × 2 tables; (3) publication in a non-English language; or (4) the reference standard was not pathology. Three studies were identified as additional related articles by checking the reference list. Ultimately, 18 studies including 969 patients were included in the present analysis (3, 11–16, 18, 19, 22–30). Figure 1 provides an overview of the study search and selection.

### Characteristics of Included Studies

The characteristics of the included studies are summarized in Table 1. The number of patients enrolled in the included studies ranged from 20 to 170. In 13 studies, patients who achieved a pathological complete response (pCR, no invasive cancer, but ductal carcinoma *in situ* may be present) were classified as pathological responders, and patients who did not achieve pCR were non-responders. In four studies, patients who achieved pCR or near pCR (residual tumor volume < 1 cm^3^, more than 90% of tumor cells disappeared, non-measurable isolated microscopic foci of reasons for the heterogeneity or *in situ* disease) were classified as responders. In another study, both methods of defining the pathological responder mentioned above were analyzed. The percentage of responders ranged from 12.5 to 42.9%. The median age of the patients was 43.3–57 years.

**Table 1:** Characteristics of the studies included in present analysis.

	**Patient characteristics**				**Study characteristics**			**Imaging characteristics**	
**References**	**Age (years)**	**No. of patient**	**Definition of responder**	**Responder number (rate)**	**Design**	**Consecutive enrollment**	**To early predict the response?**	**Magnet strength**	**Response assessment parameters**
Tudorica et al. (3)	27–75	29	pCR	5 (17.2%)	NR	No	Yes	3.0 T	Ktrans, RECIST(LD)
Ah-See et al. (16)	29–70	28	pCR and near pCR together	11 (39.2%)	P	Yes	Yes	1.5 T	Ktrans, Kep, Ve, tumor size
O'Flynn et al. (13)	52 (32–71)	32	pCR and near pCR together	13 (40.6%)	P	Yes	Yes	3.0 T	Ktrans, Kep, Ve, tumor size
Li et al. (15)	45 (28–67)	28	pCR	11 (39.3%)	P	No	Yes	3.0 T	Kep
Hahn et al. (18)	43.3 (24–59)	78	pCR	19 (24.4%)	R	Yes	No	1.5 or 3 T	Residual breast cancer size
An and Kim (27)	51.6 (29–69)	20	pCR	3 (15.0%)	NR	Yes	No	3.0 T	RECIST 1.1
Tateishi et al. (11)	57 (43–72)	142	pCR	24 (16.9%)	P	Yes	Yes	3.0 T	Kep, RECIST 1.1
Li et al. (26)	45 (28–67)	33	pCR	12 (36.4%)	P	No	Yes	3.0 T	Ktrans, Kep, Ve, LD
Nadrljanski et al. (19)	53.2 (32–77)	66	pCR and near pCR together	27 (40.9%)	P	Yes	Yes^#^	1.5 T	RECIST 1.1 (Δ tumor volume)
Cho et al. (30)	46.4 (29–65)	48	pCR^*^	6 (12.5%)	P	Yes	Yes	3.0 T	PRM, Ktrans, Kep, Ve
Sun et al. (25)	48 (29–64)	170	pCR	34 (20.0%)	P	Yes	Yes	1.5 T	Multi-parameter MRI model
van Uden et al. (23)	51.0 (38–67)	27	pCR	8 (29.6%)	R	Yes	No	1.5 or 3 T	RECIST 1.1
Abramson et al. (29)	45 (28–60)	21	pCR	9 (42.9%)	P	No	Yes	3.0 T	lesion size, lesion enhancement
Wu et al. (24)	NR	35	pCR	12 (34.3%)	R	No	Yes	3.0 T	Enhancement map, eigenmap in tumor subregions
Bottcher et al. (28)	49.5 (35–69)	54	pCR	12 (22.2%)	NR	No	No	1.5 T	RECIST
Kim et al. (12)	45 (25–67)	39	pCR and near pCR together	12 (30.8%)	P	Yes	Yes	3.0 T	Ktrans, Kep, Ve
Drisis et al. (14)	51 (25–82)	84	pCR	16 (19.0%)	R	Yes	Yes	1.5 T	Ktrans, Ve, Dmax
Kontopodis et al. (22)	NR	35	pCR	12 (34.3%)	NR	No	Yes	3.0 T	Kep, Ve

*pCR, pathological complete response; near pCR, residual tumor volume < 1 cm^3^, more than 90% of tumor cells disappeared, non-measurable isolated microscopic foci of reasons for the heterogeneity or in situ disease*.

* *both methods of defining the pathological responder mentioned were analyzed; NR, not reported; R, retrospective; P, prospective*.

# *DCE-MRI was performed to both early predict the response of breast cancer and assess the response after NAC completion; Δ, change; Ktrans, transfer constant; Kep, rate constant; Ve, relative extravascular extracellular space; PRM, parametric response map; LD, longest diameter; RECIST, response evaluation criteria in solid tumors; Dmax, maximum diameter*.

Among the included studies, ten were prospective, four were retrospective, and the other four studies did not report the type of study design. In 13 studies, patients underwent DCE-MRI after 1 or 2 cycles of NAC to early predict the response of breast cancer. In four studies, DCE-MRI was performed to evaluate the response of breast cancer after NAC completion. In another study, DCE-MRI was performed to both early predict the response of breast cancer and assess the response after NAC completion. The diagnostic performance of different parameters or analysis methods of DCE-MRI, namely, the volume transfer coefficient Ktrans (contrast agent plasma/interstitial transfer rate constant), Kep (intravasation rate constant), Ve (extravascular and extracellular volume fraction), and RECIST (Response Evaluation Criteria in Solid Tumors) guidelines, were analyzed in six, seven, six and five studies, respectively. Pathology was used as a reference standard, and response evaluation by DCE-MRI was blinded to the pathological examination results in all included studies. 3-T scanners were used in 11 studies, and 1.5-T scanners were used in five studies. In another two studies 1.5- or 3-T scanners were used.

### Data Quality Assessment

**Figure 2:**
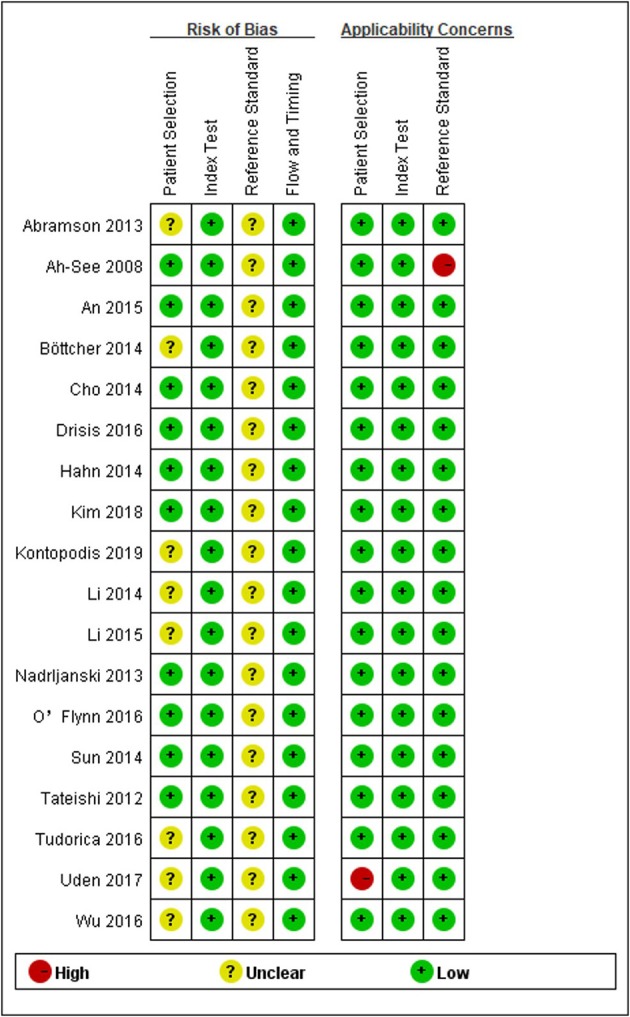
Grouped bar charts showing risk of bias **(Left)** and concerns for applicability **(Right)** for each included study using QUADAS-2.

Overall, the methodological quality of the included studies was considered acceptable, and the distribution of QUADAS-2 scores of the included studies is presented in Figure 2. In the patient selection domain, the risk of bias is unclear in the seven studies, as they did not explicitly mention whether patient enrolment was consecutive. In one study, the concern for applicability was considered high, as patients diagnosed with inflammatory breast cancer were primarily included. All studies had a low risk of bias in the index test domain. All studies had an unclear risk of bias for the reference standard domain, because it was unmentioned whether the derivation of the reference standard was blinded to DCE-MRI. All included studies had low concerns regarding applicability for this domain because postoperative pathology was used as the reference standard. All studies had a low risk of bias for flow and timing domain. No studies were excluded on the basis of quality assessment.

### Data Analysis

**Figure 3:**
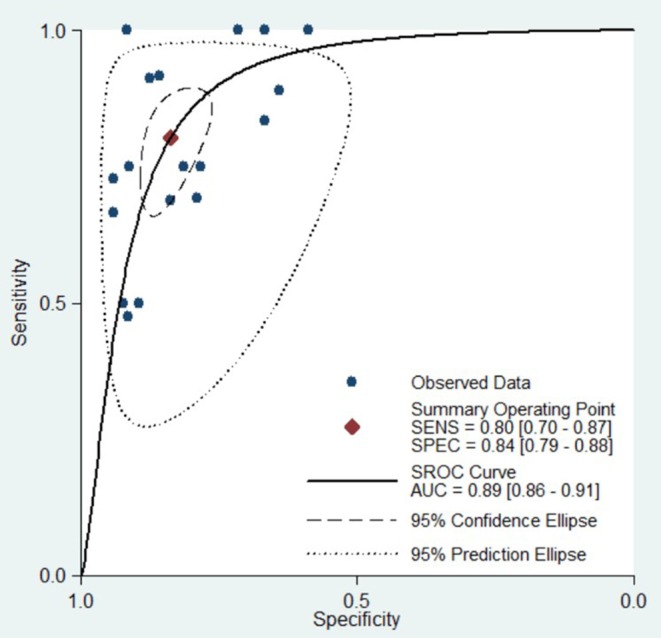
Hierarchical summary receiver operating characteristic (SROC) curve of the diagnostic performance of DCE-MRI for evaluation of the pathological response of breast cancer to neoadjuvant chemotherapy. Each solid circle represents one included study. Values in bracketsare 95% CIs. AUC, area under the curve.

For all 18 studies, the mean values and 95% CIs of pooled sensitivity, specificity, PLR, NLR, and diagnostic odds ratios (DOR) for DCE-MRI in assessing the pathological response of breast cancer to NAC were 0.80 (0.70, 0.88), 0.84 (0.79, 0.88), 4.95 (3.86, 6.35), 0.24 (0.16, 0.35), and 21.01 (13.28, 33.24), respectively (Table 2). The area under the summary receiver operating characteristic curve (AUC) was 0.89 (0.86, 0.91, Figure 3). The Cochran's Q test suggested that heterogeneity was present (*Q* = 29.37, *p* = 0.000), and the forest plots (Figure 5) also confirmed the presence of substantial heterogeneities for both sensitivity (*I*^2^ = 58.29%) and specificity (*I*^2^ = 64.54%).

**Table 2:** Results of meta-regression analysis.

**Covariates**	**Coefficient**	**SE**	***P*-value**
To early predict the response or not	0.188	0.461	0.690
Definition of responder (pCR or pCR and near pCR together)	0.501	0.502	0.334
Magnetic field (3 or 1.5-T)	−0.513	0.465	0.288
Study design (retrospective or prospective)	0.436	0.494	0.396
Response assessment parameter	−0.184	0.425	0.671

*pCR, pathological complete response; near pCR, residual tumor volume < 1 cm^3^, more than 90% of tumor cells disappeared, non-measurable isolated microscopic foci of reasons for the heterogeneity or in situ disease; SE, standard error*.

**Figure 4:**
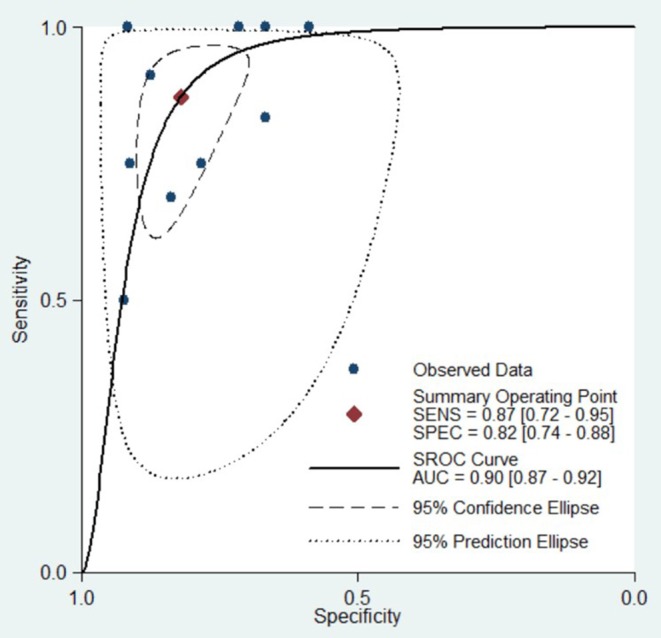
Hierarchical summary receiver operating characteristic (SROC) curve of the diagnostic performance of DCE-MRI for prediction of the pathological complete response (pCR) of breast cancer to neoadjuvant chemotherapy. Each solid circle represents one included study. Values in bracketsare 95% CIs. AUC, area under the curve.

We also performed a meta-analysis of 10 studies to analyse the diagnostic performance of DCE-MRI in the early prediction of pCR in breast cancer to NAC. The pooled sensitivity, specificity, PLR, NLR, DOR, and AUC were 0.87 (0.72, 0.95), 0.82 (0.74, 0.89), 4.86 (3.51, 6.73), 0.16 (0.07, 0.35), 30.31 (13.65, 71.82), 0.90 (0.87, 0.92), respectively (Figure 4, Table 2). The forest plots (Figure 6) showed substantial heterogeneity for sensitivity (*I*^2^ = 64.62%) and specificity (*I*^2^ = 70.04%).

**Figure 5:**
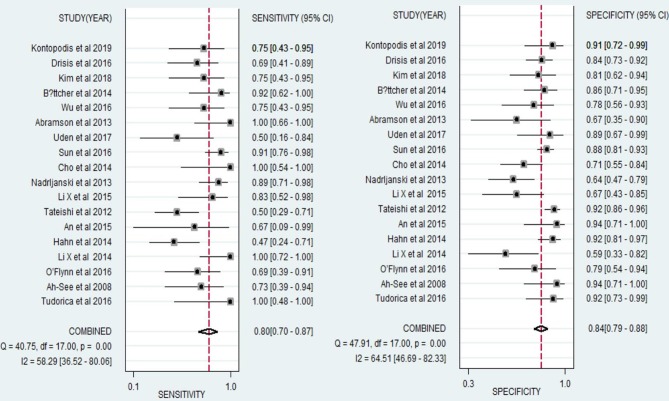
Forest plots of the sensitivity and specificity of DCE-MRI for evaluation of the pathological response of breast cancer to neoadjuvant chemotherapy. *I*^2^ > 50% indicated substantial heterogeneity in the diagnostic parameters across studies.

**Figure 6:**
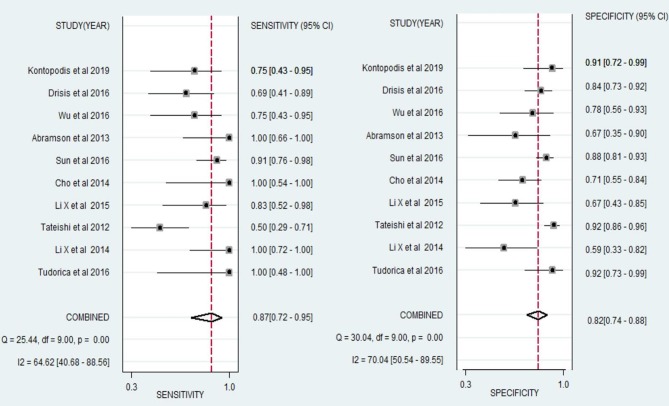
Forest plots of the sensitivity and specificity of DCE-MRI for prediction of the pathological complete response (pCR) of breast cancer to neoadjuvant chemotherapy. *I*^2^ > 50% indicated substantial heterogeneity in the diagnostic parameters across studies.

### Exploration of Heterogeneity

The results of the diagnostic threshold analysis showed that the threshold effect existed with a Spearman correlation coefficient of 0.65 and a *P*-value of 0.003.

Table 2 yields the results of the meta-regression analyses. Analysis of the covariates, namely, the time of examination, the definition of pathological responder, the magnetic field strength, the study design and the evaluation index, did not significantly affect the heterogeneity.

Table 3 shows the results of sensitivity analyses for each subgroup to explore the influence of the time of examination, the definition of pathological responder, the magnetic field strength, the study design and the evaluation index on pooled sensitivity and specificity estimates. The results of heterogeneity analysis for each subgroup are also shown in the table. Fourteen studies with the definition of responder as pCR had a higher sensitivity of 0.83 (0.67, 0.92) and an equivalent specificity of 0.85 (0.79, 0.89) compared with studies with the definition of responder as pCR and near pCR [*n* = 5, sensitivity 0.72 (0.57, 0.84), specificity 0.82 (0.68, 0.91)]. Studies (*n* = 14) using DCE-MRI to early predict the pathological response of breast cancer had a higher sensitivity (0.83 vs. 0.71) and an equivalent specificity (0.80 vs. 0.86) compared to studies (*n* = 5) that did not use DCE-MRI for early prediction. Studies setting RECIST (*n* = 5), Kep (*n* = 6), and Ktrans (*n* = 7) as the response assessment parameters had good diagnostic performance with AUC values of 0.85 (0.82, 0.88), 0.83 (0.79, 0.86), and 0.80 (0.76, 0.83), respectively, but Ve (*n* = 6) had moderate diagnostic performance with an AUC value of 0.71 (0.67, 0.75). The pooled sensitivity and specificity of studies that used a 3.0-T device or a 1.5-T device were equivalent.

**Table 3:** Sensitivity analyses performed for subgroups of studies.

**Analysis**	**No. of study**	**Sensitivity (%)**	**Specificity (%)**	**PLR (%)**	**NLR (%)**	**DOR (%)**	**AUC (%)**	***I*^**2**^**
Overall	18	0.80 (0.70, 0.88)	0.84 (0.79, 0.88)	4.95 (3.86, 6.35)	0.24 (0.16, 0.35)	21.01 (13.28, 33.24)	0.89 (0.86, 0.91)	93.19%
To early predict the pCR of BC to NAC using DCE-MRI	10	0.87 (0.72, 0.95)	0.82 (0.74, 0.89)	4.86 (3.51, 6.73)	0.16 (0.07, 0.35)	30.31 (13.65, 71.82)	0.90 (0.87, 0.92)	90.09%
**To early predict the response?**								
Yes	14	0.83 (0.74, 0.90)	0.80 (0.72, 0.87)	4.23 (3.02, 5.91)	0.21 (0.13, 0.33)	20.41 (11.99, 34.76)	0.89 (0.86, 0.91)	94.72%
No	5	0.71 (0.49, 0.87)	0.86 (0.72, 0.93)	5.00 (2.80, 8.87)	0.34 (0.18, 0.62)	14.72 (7.12, 30.4)	0.87 (0.83, 0.89)	72.0%
**Definition of responder**								
pCR	14	0.83 (0.67, 0.92)	0.85 (0.79, 0.89)	5.46 (4.17, 7.14)	0.20 (0.11, 0.38)	27.13 (13.97, 52.67)	0.90 (0.87, 0.92)	92.32%
pCR and near pCR together	5	0.72 (0.57, 0.84)	0.82 (0.68, 0.91)	4.00 (2.30, 6.95)	0.34 (0.22, 0.53)	11.83 (5.51, 25.41)	0.84 (0.81, 0.87)	67.66%
**Magnetic field**								
3.0-T	11	0.82 (0.68, 0.91)	0.81 (0.73, 0.87)	4.39 (3.16, 6.10)	0.22 (0.12, 0.40)	19.75 (10.07, 38.72)	0.88 (0.85, 0.91)	89.87%
1.5-T	5	0.85 (0.75, 0.91)	0.84 (0.75, 0.90)	5.23 (3.33, 8.21)	0.18 (0.11, 0.31)	28.79 (13.57, 61.08)	0.91 (0.88, 0.93)	0%
**Study design**								
Prospective	10	0.86 (0.74, 0.93)	0.80 (0.70, 0.86)	4.20 (2.97, 5.93)	0.18 (0.09, 0.33)	23.74 (12.10, 46,57)	0.89 (0.86, 0.92)	92.11%
Retrospective	4	0.61 (0.45, 0.74)	0.86 (0.79, 0.92)	4.49 (2.85, 7.09)	0.46 (0.31, 0.66)	9.86 (4.83, 20.15)	0.84 (0.81, 0.87)	0%
**Response assessment parameter**								
Ktrans	6	0.72 (0.59, 0.83)	0.78 (0.64, 0.88)	3.33 (1.77, 6.24)	0.35 (0.21, 0.59)	9.44 (3.25, 27.43)	0.79 (0.76, 0.83)	0%
Kep	7	0.76 (0.54, 0.89)	0.76 (0.62, 0.86)	3.20 (2.16, 4.74)	0.32 (0.17, 0.60)	10.11 (4.90, 20.82)	0.83 (0.79, 0.86)	92.86%
Ve	6	0.42 (0.24, 0.63)	0.89 (0.71, 0.96)	3.79 (1.62, 8.86)	0.65 (0.48, 0.88)	5.84 (2.27, 15.01)	0.71 (0.67, 0.75)	83.37%
RECIST	5	0.70 (0.45, 0.86)	0.83 (0.74, 0.89)	4.04 (2.87, 5.70)	0.37 (0.19, 0.70)	10.96 (5.00, 24.01)	0.85 (0.82, 0.88)	82.12%

*pCR, pathological complete response; near pCR, residual tumor volume < 1 cm^3^, more than 90% of tumor cells disappeared, non-measurable isolated microscopic foci of reasons for the heterogeneity or in situ disease; Ktrans, transfer constant; Kep, rate constant; Ve, relative extravascular extracellular space; RECIST, response evaluation criteria in solid tumors; BC, breast cancer; NAC, neoadjuvant chemotherapy; DCE-MRI, dynamic contrast-enhanced magnetic resonance imaging; PLR, positive likelihood ratio; NLR, negative likelihood ratio; DOR, diagnostic odds ratios; AUC, the area under the curve; %, 95% confidence intervals; I^2^, the inconsistency index*.

**Figure 7:**
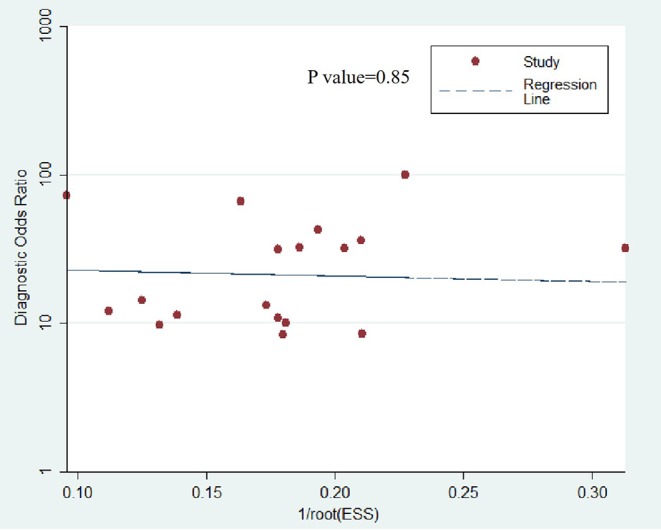
Deeks funnel plot shows the likelihood of publication bias is low with a *P* value of 0.85. ESS, effective sample size.

The Deeks et al. (31) funnel plot (*P* = 0.89) is presented in Figure 7, which suggests there was no publication bias.

## Discussion

**Figure 8:**
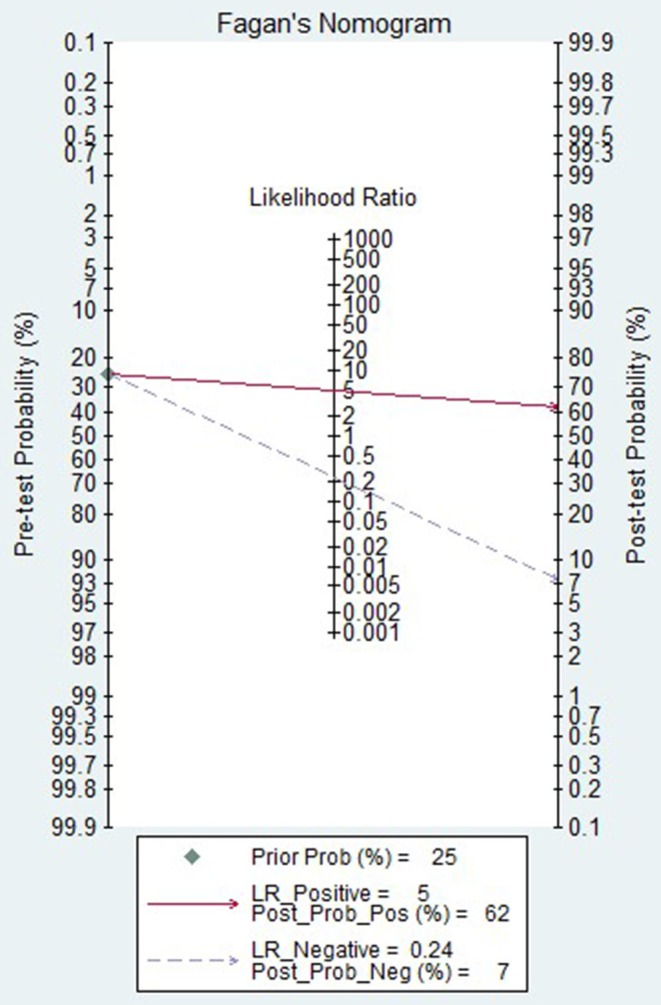
Fagan plot of the DCE-MRI test for evaluation of the pathological response of breast cancer to neoadjuvant chemotherapy.

Neoadjuvant chemotherapy, which started before breast cancer surgery, was introduced in the 1970s, aiming to downstage locally advanced (inoperable) breast cancer and make it operable (32). A previous meta-analysis published in Lancet Oncology demonstrated that NAC results in higher rates of breast-conserving therapy than does adjuvant chemotherapy, without compromising distant recurrence, breast cancer survival, or overall survival (33). Appropriate evaluation of the efficacy of NAC can not only guide the treatment plan of patients but also evaluate the response of tumors to drugs before surgery to provide a reliable theoretical basis for postoperative treatment. In the present meta-analysis, we evaluated the diagnostic accuracy of DCE-MRI for evaluating the pathological response of breast cancer to NAC. Our results indicated that the pooled sensitivity and specificity of the 18 studies included were 0.80 (95% CI 0.70, 0.88) and 0.84 (95% CI 0.79, 0.88). Based on this good diagnostic performance, DCE-MRI could be considered an important auxiliary method for evaluating the pathological response of breast cancer to NAC. In the present study, the likelihood ratio and post-test probability were both moderate (Figure 8). Use of a DCE-MRI test would raise the post-test probability to 62 from 25% with a PLR of 5 when the pretest was positive and would reduce the post-test probability to 7% with an NLR of 0.24 when the pretest was negative. This indicates that DCE-MRI was helpful for increasing the accuracy of evaluating the pathological response of breast cancer to NAC.

Substantial heterogeneity among the included studies was detected. Based on meta-regression analyses, the covariates, namely, the time of examination, the definition of pathological responder, the magnetic field strength, the study design and the evaluation index, showed no significant factors affecting the heterogeneity. Thus, the heterogeneity could not be explained by meta-regression analysis.

The definition of a pathological responder is an important factor when assessing the diagnostic performance of DCE-MRI in evaluating the pathological response of breast cancer to NAC. Although it not significant according to the meta-regression analysis, sensitivity analysis showed that studies setting pCR (*n* = 14) as a responder showed a tendency for higher sensitivity compared with those setting pCR and near pCR together (*n* = 5) as a responder (0.83 vs. 0.72). We speculate that it is easier to evaluate pCR on the images because the morphological and haemodynamic changes of the lesions are more typical and notable. Therefore, setting pCR as a responder may improve the diagnostic sensitivity of DCE-MRI. In a previous study (30) published in the journal Radiology in 2014, Cho et al. studied DCE-MRI in the early prediction of pathological responses to NAC in 48 breast cancer patients using a parametric response map (PRM) analysis. Their research also showed a higher sensitivity (100%, six of six pCR) but a lower specificity (71%, 30 of 42 npCR) in the prediction of pCR compared with the prediction of a good response, which yielded 55% (21 of 38 good responses) sensitivity and 90% (nine of 10 minor responses) specificity. However, more controlled clinical studies are needed to confirm this conclusion in the future.

Another aspect of the clinical setting is the timing of the response assessment. The earlier the response state of patients to NAC is obtained, the better for the patients. In the present meta-analysis, 14 studies evaluated the performance of DCE-MRI in the early prediction of the response to NAC in breast cancer. Based on sensitivity analysis, studies (*n* = 14) using DCE-MRI to early predict (assess the response after one or two cycles of NAC) the pathological response of breast cancer had a higher sensitivity (0.83 vs. 0.71) and equivalent specificity (0.80 vs. 0.86) compared to studies (*n* = 5) that did not use DCE-MRI to early predict (assess the response after NAC completion). We speculate that morphological and haemodynamic changes of the lesions on DCE-MRI after 1 ~ 2 cycles of NAC treatment may provide important reference information to early predict the lesion response to treatment. A previous study (19) found similar results. In this study, the sensitivity and specificity for responder identification after the second cycle of NAC, based on pure morphokinetic features on DCE-MRI examination, were 93 and 40%, respectively, and upon the completion of NAC, they were 87 and 63%, respectively. A meta-analysis of 10 studies using DCE-MRI for the early prediction of pCR in breast cancer to NAC also provided good diagnostic accuracy for DCE-MRI (sensitivity 0.87 and specificity 0.82, respectively) in the present study. These results verified that DCE-MRI also had a good diagnostic performance in the prediction of response in the early stage of NAC compared with the evaluation of response after NAC.

Different assessment criteria or parameters of DCE-MRI were used to evaluate the response of breast cancer to NAC in the included studies. So, we also performed a meta-analysis to evaluate the diagnostic accuracy of these criteria or parameters of DCE-MRI. Our results demonstrated that studies using RECIST (*n* = 5), Kep (*n* = 6), and Ktrans (*n* = 7) as response assessment criteria or parameters had good diagnostic performance with AUC values of 0.85 (0.82, 0.88), 0.83 (0.79, 0.86), and 0.79 (0.76, 0.83), respectively, but Ve (*n* = 6) had moderate diagnostic performance with an AUC value of 0.71 (0.67, 0.75) and a low sensitivity of 0.42 (0.24, 0.63). We are not surprised by these results. The quantitative parameters of DCE-MRI, namely, Ktrans, Kep, Ve, provide information about the microcirculation of tumors and are indicative of malignant-grade tumors. Ktrans and Kep can directly reflect the permeability of tumor capillaries. Studies (12, 16) have indicated that the Ktrans and Kep values of breast cancer patients who accept neoadjuvant chemotherapy were significantly correlated with pathological response. Ve is related to the active cell environment and is characterized by the compactness of extracellular space outside the blood vessels. Tofts et al. (34) reported that the Ve value is unstable, which may be related to the influence of oedema around lesions. This may be one of the potential factors for the low sensitivity of Ve in assessing the pathological response of breast cancer to NAC.

A recent meta-analysis (8) of 54 studies encompassing 5,272 patients analyzed the diagnostic performance of CE-MRI for evaluating the pathological complete response (pCR) of breast cancer to NAC, and the pooled sensitivity and specificity were 0.64 (95% CI, 0.56–0.70) and 0.92 (95% CI, 0.89–0.94), respectively. In our present analysis, we obtained an improved sensitivity (0.83 vs. 0.64) but an equivalent specificity (0.85 vs. 0.92) of DCE-MRI for assessing the pCR of breast cancer to NAC. This result may indicate that DCE-MRI has better diagnostic sensitivity than CE-MRI. DCE-MRI can not only provide morphological characteristics but also haemodynamic and quantitative information about the lesion. Most studies in previous meta-analysis used a 1.5-T (73.7%) scanner; however, in the present meta-analysis, most studies (11/18) used a 3.0-T scanner. A high magnetic field can improve image resolution, which may contribute to improving the diagnostic sensitivity of MRI for evaluating the pCR of breast cancer to NAC.

Exploration of heterogeneity was an indispensable part of meta-analysis when analyzing the pooled results. Introducing improper heterogeneity will decrease the credibility of the findings (35). The substantial heterogeneity among the studies was detected in present meta-analysis. The included studies are different in many ways: the studies' primary objectives (to early predict the response or not), definition of pathological responder, magnetic field strength, study design and response assessment parameter used. Although study characteristics mentioned above not being significant factors in the meta-regression analysis, these differences could still ultimately lead to unknown biases. Moreover, further unmentioned differences between studies may be the cause of heterogeneity. To assess the influence of study characteristics mentioned above on results and heterogeneity, we performed sensitivity analyses for the relevant covariates. The results of the sensitivity analysis showed that the heterogeneity of some subgroups was significantly reduced, but the heterogeneity of most subgroups was still significant. Of the 18 studies included, 10 studies analyzed the diagnostic performance of DCE-MRI in the early prediction of the pathological complete response (pCR) in breast cancer to NAC. We also performed a meta-analysis on these 10 relatively homogeneous studies. The results showed a reduction in heterogeneity, but still significant. We will update our study in the future, if more homogeneous studies can be included. The diagnostic threshold analysis showed the presence of the threshold effect in the present meta-analysis. Threshold effects are an important source of diagnostic meta-analysis heterogeneity. The DCE-MRI criteria or parameters and cut-off points used to evaluate the pathological response of NAC were not standardized in the included studies, leading to the threshold effect. When a threshold effect exists, sensitivity and specificity are negatively correlated. Therefore, in this analysis, the SROC curve was also used to calculate the AUC to evaluate the diagnostic performance of DCE-MRI.

## Limitations

There are several limitations in the present meta-analysis. The main limitation of this analysis was that the presence of substantial heterogeneity among the studies and threshold effect in the present meta-analysis were detected. Although study characteristics not being significant factors in the meta-regression, these differences could still ultimately lead to unknown biases. Selecting more homogeneous articles for inclusion in this analysis may solve this problem, but it can lead to selection bias. The second limitation may be the small number of studies included, which prevents us from investigating all possible heterogeneity reasons. We did not perform subgroup analysis for different subtypes of breast cancer because only two studies had analyzed the diagnostic accuracy of DCE-MRI for evaluating the pathological response to NAC in different subtypes of breast cancer, which needs to be elucidated in future studies. Another limitation was that the risk of bias for all included studies was unclear because no information is provided as to whether the reference standard was blinded to DCE-MRI results. Fortunately, the diagnostic accuracy would not be overestimated, for in all included studies, the index test was blinded to the reference standard. Therefore, this unclear risk may not have had a significant effect on the outcomes.

## Conclusion

Our results indicated that DCE-MRI could be considered an important auxiliary method for evaluating the pathological response of breast cancer to NAC and used as an effective method for dynamically monitoring the efficacy during NAC. DCE-MRI also performed well in predicting the pCR of breast cancer to NAC. However, due to the heterogeneity of the included studies, caution should be exercised in applying our results.

## Author Contributions

CS and LL contributed conception and design of the study. QC, JH, and MM contributed to collection and assembly of data. JH and KY performed data analysis and interpretation. QC wrote the first draft of the manuscript. JL revised the language and reviewed the manuscript. All authors read and approved the final version.

### Conflict of Interest

The authors declare that the research was conducted in the absence of any commercial or financial relationships that could be construed as a potential conflict of interest.
